# Tracking the Influence of Predictive Cues on the Evaluation of Food Images: Volatility Enables Nudging

**DOI:** 10.3389/fpsyg.2020.569078

**Published:** 2020-09-15

**Authors:** Kajornvut Ounjai, Lalida Suppaso, Jakob Hohwy, Johan Lauwereyns

**Affiliations:** ^1^Biological Engineering Program, Faculty of Engineering, King Mongkut’s University of Technology Thonburi, Bangkok, Thailand; ^2^School of Philosophical, Historical, and International Studies, Monash University, Melbourne, VIC, Australia; ^3^Graduate School of Systems Life Sciences, Kyushu University, Fukuoka, Japan; ^4^School of Interdisciplinary Science and Innovation, Kyushu University, Fukuoka, Japan; ^5^Faculty of Arts and Science, Kyushu University, Fukuoka, Japan

**Keywords:** predictive dissonance, nudging, volatility, evaluative processing, naturalistic food images

## Abstract

In previous research on the evaluation of food images, we found that appetitive food images were rated higher following a positive prediction than following a negative prediction, and vice versa for aversive food images. The findings suggested an active confirmation bias. Here, we examine whether this influence from prediction depends on the evaluative polarization of the food images. Specifically, we divided the set of food images into “strong” and “mild” images by how polarized (i.e., extreme) their average ratings were across all conditions. With respect to the influence from prediction, we raise two alternative hypotheses. According to a predictive dissonance hypothesis, the larger the discrepancy between prediction and outcome, the stronger the active inference toward accommodating the outcome with the prediction; thus, the confirmation bias should obtain particularly with strong images. Conversely, according to a nudging-in-volatility hypothesis, the active confirmation bias operates only on images within a dynamic range, where the values of images are volatile, and not on the evaluation of images that are too obviously appetitive or aversive; accordingly, the effects from prediction should occur predominately with mild images. Across the data from two experiments, we found that the evaluation of mild images tended to exhibit the confirmation bias, with ratings that followed the direction given by the prediction. For strong images, there was no confirmation bias. Our findings corroborate the nudging-in-volatility hypothesis, suggesting that predictive cues may be able to tip the balance of evaluation particularly for food images that do not have a strongly polarized value.

## Introduction

In the last decade, theoretical accounts of brain and mind have pulled predictive processing to the foreground as a core aspect in many, if not all, of the functions traditionally studied in psychology, including perception, memory, attention, learning, and decision making. Bayesian inference, active inference, and discussions of prediction error minimization prove to be powerful tools, not only in modeling human behavior, but also in understanding the underlying neural mechanisms (Friston et al., 2013; Fitzgerald et al., 2015; Hohwy, 2017; Rigoli et al., 2017; Wagenmakers et al., 2018a, b). This theoretical approach with a dominant role for prediction derives principally from earlier, empirical work on the neural correlates of reward and decision making (reviewed in Schultz, 2015; see also D’Astolfo and Rief, 2017; O’Doherty et al., 2017). The archetypal finding, although not entirely unchallenged, is that the activity of dopamine neurons corresponds to reward prediction error. A positive prediction error, say, an unexpected reward, is associated with an increase in dopamine activity, whereas a negative prediction error is associated with a decrease in dopamine activity. However, a positive prediction error may not necessarily be associated with a positive affect, given the dissociation between “wanting versus liking” and the complexity of pleasure systems in the brain (Berridge and Kringelbach, 2015).

Similarly, it is unclear whether or how the prediction errors may influence concurrent evaluative processing. Take a value-based judgment task with tricks or treats. How would a predictive cue influence the subsequent evaluation, particularly if the outcome does not match with the prediction? Will we give a higher evaluation to a surprise treat, or to an expected treat? Considering the concept of dopamine prediction error and its complex connection to affect, it is not immediately clear how the influence of the prior should play out. On the one hand, one might point out that the predictive cue should generate an initial dopamine prediction error, activating a positive or negative anticipation that could bias the subsequent evaluation. On the other hand, in case of a violation of the prediction, the trick or treat should itself generate a second dopamine prediction error, which could override the first signal and affect the evaluation of the presented item. Would we see bias in line with the prediction (higher ratings for expected treats) or an opposite effect (higher ratings for surprise treats)? In our previous study, we asked this question empirically, using an evaluation task with a bivalent set of food images (Ounjai et al., 2018). Our data showed that appetitive food images were rated higher following a positive prediction than following a negative prediction, and vice versa for aversive food images. Thus, the evaluations tended to be biased in the direction of the prediction.

In our previous study (Ounjai et al., 2018), analysis of the reaction times further showed that valid predictions (e.g., a positive prediction followed by an appetitive food image) produced faster evaluations than invalid predictions. Moreover, during the waiting period between the predictive cue and the target image, we observed gaze biases indicative of a preparatory process in line with the prediction. For instance, with a continuous rating scale from −10 on the left to +10 on the right, the subject’s gaze tended to be biased to the right half of the screen following a positive cue, even before the appearance of the food image. Such gaze biases suggested that the influence from the predictive cue depended on active, voluntary processing. The entire pattern of data was interpreted as an active confirmation bias, by which subjects effectively used the predictive cues to guide their subsequent evaluative processing toward confirming what is expected. This finding added to a growing set of studies tracking self-reinforcing expectancy effects (Jepma et al., 2018) and the integration of context into evaluation (Schmidt et al., 2017).

Here, we take a closer look at this type of confirmation bias by considering the level of discrepancy between prediction and outcome. For instance, following a negative cue, the presentation of a strongly appetitive image would reflect a larger expectation violation than the presentation of a moderately appetitive image. We use the term “polarization” to indicate the extent to which the ratings tend toward the polar extremes–either extremely positive or extremely negative–the more extreme, the more polarized (see, e.g., Askarisichani et al., 2019, for a similar usage of the term “polarization”).

Importantly, the literature on expectation violations appears to imply conflicting views on how the size of the violation might affect subsequent evaluative processing. According to a theory of predictive dissonance (Kaaronen, 2018), the processing following expectation violations can be compared with the active efforts toward the reduction of cognitive dissonance (Festinger, 1957). Stronger efforts would be required with greater magnitude of dissonance. One approach to dissonance reduction is by selectively harvesting sensory information, which is consonant with our predictions–the “dark side” of this would be confirmation bias (Clark, 2016). Applying these ideas to the current context, we derive a predictive dissonance hypothesis by which the confirmation bias–or the tendency to stick with the prediction despite the outcome–should be most active with highly polarized food images, leading to strong influences from the predictive cue.

Alternatively, several studies have emphasized that volatility is a critical factor in value updating (Massi et al., 2018; Findling et al., 2019). Volatility, here, would pertain to the affective values of various stimuli. Strongly appetitive or strongly aversive images should have less volatile affective values, consistently eliciting very polarized evaluations. Instead, moderately appetitive or aversive stimuli may generate more imprecision in subjects’ evaluations, sometimes liked or disliked, more susceptible to subjective differences. The predictive cues would then be more influential for stimuli with volatile affective values (i.e., moderately appetitive or aversive) than for stimuli with stable affective values (i.e., strongly appetitive or aversive). Thus, the predictions might tip–or nudge–the balance one way or another only within a dynamic range for food images whose value is less salient. Analogous to the concept of nudging in behavioral economics (Thaler and Sunstein, 2008; see also Noggle, 2018), we label this hypothesis as “nudging in volatility.”

Concretely, reanalyzing our previous data (Ounjai et al., 2018), we investigate in the present study how the confirmation bias operates with strong versus mild images. According to the predictive dissonance hypothesis, the confirmation bias should be more pronounced for strongly polarized food images than for less polarized food images. Thus, for strong positive images, the ratings should be higher after positive cues than after negative cues; for strong negative images, the ratings should be lower after negative cues than after positive cues. These cue validity effects would be less pronounced with mild images.

According the nudging-in-volatility hypothesis, for mild positive images, the ratings should be higher after positive cues than after negative cues; for mild negative images, the ratings should be lower after negative cues than after positive cues. These cue validity effects would be less pronounced with strong images.

## Methods

The current study is based on a reanalysis of the data set that was published previously by Ounjai et al. (2018). For present purposes, we considered only the manual joystick responses with the evaluative ratings, particularly in trials with positive or negative predictive cues that could produce expectation violations.

### Subjects

In Experiment 1, there were 42 subjects. All were Kyushu University students (26 male and 16 female subjects) with a mean age of 22.45 ± 3.63 years. In Experiment 2, there were 66 subjects. All were Kyushu University students (38 male and 28 female subjects) with a mean age of 23.94 ± 4.54 years. In both experiments, all subjects had normal or corrected-to-normal vision. All subjects gave informed consent and reported that they were in healthy condition before and after the experiment.

### Apparatus and Stimuli

The visual stimuli were presented in a dimly lit room on a 23.8-inch full-high-definition flat-panel monitor, with a display resolution of 1,920 × 1,080 pixels. The subjects were seated approximately 62 cm from the monitor. To minimize head movement, a chin rest with a forehead support was used. The evaluation responses were recorded using a joystick (Logitech, Switzerland; model no. 963290-0403). All visual stimuli were presented as inset images on a white background in the middle of the otherwise black screen. The size of the inset image was fixed at 380 × 380 pixels for the predictive cues, and at 600 × 600 pixels for the food images. The predictive cues were icons: a tray for positive and a hazard sign for negative. We also used a checkerboard for neutral cue. Food images were drawn from the FoodCast research image database (FRIDa), developed by Foroni et al. (2013). This database was supplemented with non-copyrighted images to construct a set of 200 food images with a balanced range of appetitive and aversive stimuli.

### Experimental Procedures

#### Experiment 1

Participants were asked to evaluate 180 naturalistic food images in three consecutive blocks of 60 trials. At the start of each trial, the word “short” or “long” was presented for 1 s in the middle of the screen to indicate the delay time between the predictive cue and the target image, either 1 or 9 s. Next the predictive cue was shown at the center of the screen for 1 s, followed by the blank screen for the delay period. Then, the target image was shown for 2 s and in turn replaced by the response screen. The subject had maximally 6 s to evaluate the food image by bending the joystick to move the cursor on the evaluation bar from −10 to 10. The bending angle was used to indicate the evaluation score. After the response was made, there was a blank screen for 2 s as intertrial interval.

Different icons were used for the predictive cues to indicate the outcome, either appetitive or aversive, whereas the color indicated the reliability level of the cue, either 100% certain or 50% uncertain. Here, 50% positive predictive cues refer to food-tray cues, whereas 50% negative cues refer to danger-sign cues. Rationally speaking, these predictive cues were uninformative; however, we reasoned that learned associations might elicit “framing effects” (i.e., cognitive biases). Accordingly, this also raises an important limitation of the present (and our previous) study. These predictive cues were not visually neutral.

The color assignment was counterbalanced across subjects. The reliability of the predictive cue was further indicated numerically in percentage, presented in small print beneath the icon.

The evaluations had to be given on a continuous rating scale from −10 to 10, with a value of −10 for a maximally disgusting food image and a value of +10 for a maximally attractive food image.

The experiment included 180 trials, consisting of 15 repetitions of each of the 12 conditions, with three levels of cue type (certain, valid, invalid), two levels of outcome type (positive and negative), and two levels of delay time (1 s and 9 s). No food image was presented more than once. The 180 trials were presented in pseudorandom order to ensure that each block of 60 trials contained five repetitions of each condition.

For present purposes, for Experiment 1, we analyzed only the trials with potential expectation violations; thus, we analyzed only the trials with 50% positive or negative predictive cues. Additionally, because there were no effects of delay, we pooled the data of the 1-s and 9-s delay trials.

#### Experiment 2

Participants were asked to evaluate 200 naturalistic food images in four consecutive blocks of 50 trials. The procedures were the same as in Experiment 1 except for the following. The delay time between the predictive cue and the food image was fixed at 2 s, and no word cue was given to indicate the delay time at the beginning of the trial. The cue reliability for the positive and negative cues was fixed at 75%, and a third type of cue was included (a neutral cue, represented by a checkerboard as icon).

The evaluation bar assignment was changed for two groups of subjects, with either a conventional alignment (negative–left, positive–right) or the opposite alignment. Here, subjects were asked to confirm their evaluation by clicking the trigger on the joystick.

The experiment included 200 trials, divided into six conditions, with three levels of cue type (valid 75%, neutral, invalid 25%) and two levels of outcome type (positive and negative). The valid 75% conditions consisted of 60 repetitions, whereas the invalid 25% and the neutral conditions each consisted of 20 repetitions. No food image was presented more than once. The 200 trials were presented in pseudorandom order to ensure that each block of 50 trials contained the same distribution of trials per condition.

For present purposes, for Experiment 2, we analyzed only the trials with positive or negative predictive cues; thus, we analyzed only the valid 75% and invalid 25% conditions.

### Analysis

The dependent measure of interest in the current study was the evaluative ratings on a scale from −10 to +10. In order to facilitate the comparison across appetitive and aversive images, we flipped the sign of the evaluations so that all ratings (most appetitive as well as most aversive) tended toward +10. In order to examine the effects of the predictive cues as a function of the intrinsic polarization of the images, we separated “strong” from “mild” images via a median split based on the overall average ratings of the food images collapsed across all conditions and all subjects (i.e., based on the data in Figure 1 of Ounjai et al., 2018). Then, separately for each experiment, we performed a three-way within-subjects repeated-measures analysis of variance (ANOVA) on the average ratings of the food images. In this analysis, the three within-subjects factors were valence (appetitive or aversive images), polarization (strong or mild images), and validity (valid prediction or invalid prediction). To address the present research question directly, separately for each experiment, we performed planned contrasts, using paired *t*-tests, pooled across appetitive and aversive images. For each subject, we calculated the validity effects (valid minus invalid) separately for strong images and for mild images.

Finally, to assess the overall strength of the evidence for or against the predictive dissonance hypothesis and the nudging-in-volatility hypothesis, we conducted Bayesian testing as follows. Pooled across both experiments and pooled across appetitive and aversive images, we computed for each subject the validity effects (valid minus invalid) for strong images and for mild images. Then, we performed Bayesian paired *t*-testing on the validity effect measures for strong versus mild images, with subjects as degrees of freedom. This was done with two different alternative hypotheses: in the first test, the null hypothesis was pitched against the predictive dissonance hypothesis (implying that strong images should yield larger validity effect measures than mild images); in the second test, the null hypothesis was pitched against the nudging-in-volatility hypothesis (implying that strong images should yield smaller validity effect measures than mild images). The Bayesian testing was conducted following the guidelines and using the JASP software package provided by Wagenmakers et al. (2018a, b).

## Results

**FIGURE 1:**
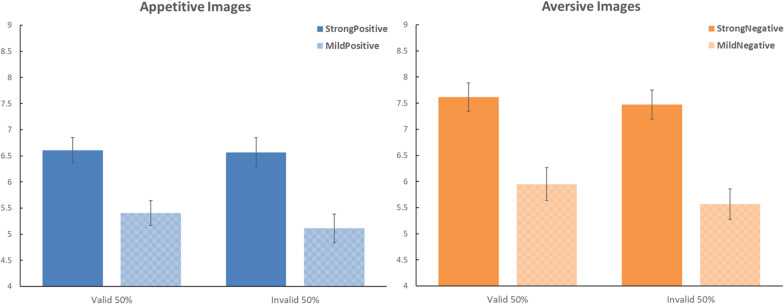
Effects of predictive cues on the evaluation of food images in Experiment 1. The **left panel** shows the data for the appetitive images. The **right panel** shows the data for the aversive images. Dark blue and dark orange bars present the data for the strong images; the light-shaded bars present the data for the mild images. On the horizontal axes, the bars are separated as a function of the validity of the predictive cues. Error bars represent the standard error of the mean. In this experiment, the framing by the predictive cues did not correlate with the outcome; there was an equal probability for the cue to be valid (i.e., matching valence with the outcome) or invalid (i.e., mismatching valence with the outcome).

The mean ratings for Experiment 1 are presented in Figure 1 as a function of the valence of the images (appetitive or aversive), the polarization (strong versus mild), and the validity of the cue–target relationship (valid or invalid). The three-way ANOVA produced significant main effects of the valence of the images, *F*(1,41) = 5.279, Mean Squared Error (MSE) = 8.474, η_*p*_^2^ = 0.114, *p* < 0.05, and the polarization of the images, *F*(1,41) = 149.696, MSE = 1.362, η_*p*_^2^ = 0.785, *p* < 0.001. The main effect of the validity of the cue–target relationship was not significant, *F*(1,41) = 2.867, MSE = 1.346, η_*p*_^2^ = 0.065, *p* = 0.098. The interaction between validity and polarization was significant, *F*(1,41) = 6.963, MSE = 0.626, η_*p*_^2^ = 0.145, *p* < 0.05. The other interactions were not significant: valence by cueing [*F*(1,41) = 0.432, MSE = 0.441, η_*p*_^2^ = 0.010, *p* = 0.515], polarization by cueing [*F*(1,41) = 1.668, MSE = 0.769, η_*p*_^2^ = 0.039, *p* = 0.204], or the three-way interaction [*F*(1,41) = 0.001, MSE = 0.589, η_*p*_^2^ = 0.001, *p* = 0.979].

To address our present research question directly, we conducted planned contrasts for the validity effects separately for strong images and mild images. For strong images, valid cues yielded a ratings average of 7.111 (SD = 1.325), whereas invalid cues yielded a ratings average of 7.020 (SD = 1.435); this validity effect was not significant, *t*(41) = 0.816, *p* = 0.419, Cohen *d* = 0.126. For mild images, valid cues yielded a ratings average of 5.677 (SD = 1.447), whereas invalid cues yielded a ratings average of 5.339 (SD = 1.356); this validity effect was not significant, *t*(41) = 1.734, *p* = 0.091, Cohen *d* = 0.267.

**FIGURE 2:**
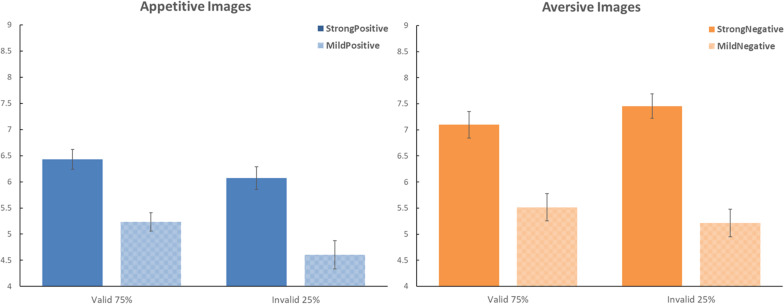
Effects of predictive cues on the evaluation of food images in Experiment 2. Same format as in Figure 1. In this experiment, the framing by the predictive cues correlated with the outcome; there was a 75% probability for the cue to be valid (i.e., matching valence with the outcome) versus 25% probability for the cue to be invalid (i.e., mismatching valence with the outcome).

The mean ratings for Experiment 2 are presented in Figure 2 in the same format as in Figure 1. The three-way ANOVA produced significant main effects of the valence of the images, *F*(1,65) = 8.358, MSE = 8.553, η_*p*_^2^ = 0.114, *p* < 0.01, and the polarization of the images, *F*(1,65) = 281,662, MSE = 1.231, η_*p*_^2^ = 0.812, *p* < 0.001. The main effect of the validity of the cue–target relationship was not significant, *F*(1,65) = 3.092, MSE = 2.317, η_*p*_^2^ = 0.045, *p* = 0.083. There were significant interactions between validity and polarization, *F*(1,65) = 12.566, MSE = 0.557, η_*p*_^2^ = 0.162, *p* < 0.001; between validity and valence, *F*(1,65) = 9.654, MSE = 0.949, η_*p*_^2^ = 0.129, *p* < 0.005; and between polarization and valence, *F*(1,65) = 14.068, MSE = 0.793, η_*p*_^2^ = 0.178, *p* < 0.001. The *F* value for the three-way interaction was not significant, *F*(1,65) = 2.270, MSE = 0.562, η_*p*_^2^ = 0.034, *p* = 0.137.

Again, as in Experiment 1, we conducted planned contrasts in Experiment 2 to examine the validity effects separately for strong images and mild images. For strong images, valid cues yielded a ratings average of 6.764 (SD = 1.541), whereas invalid cues yielded a ratings average of 6.762 (SD = 1.442); this validity effect was not significant, *t*(65) = 0.021, *p* = 0.983, Cohen’s *d* = 0.002. For mild images, valid cues yielded a ratings average of 5.374 (SD = 1.441), whereas invalid cues yielded a ratings average of 4.911 (SD = 1.628); this validity effect was significant, *t*(65) = 2.764, *p* < 0.01, Cohen *d* = 0.340. Thus, in Experiment 2, there was a significant confirmation bias only for mild images.

Finally, Bayesian testing provided overall estimates of the strengths of the evidence for or against the present hypotheses under investigation, based on the data pooled across both experiments. The descriptive statistics for the sample of 108 subjects showed a mean of 0.037 (SD 0.906) for the validity effect measures with strong images versus a mean of 0.415 (SD 1.320) for the validity effect measures with mild images.

**FIGURE 3:**
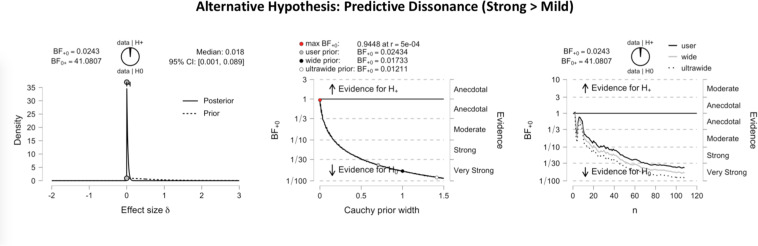
Inferential plots of Bayesian paired-samples *t*-test, with predictive dissonance as the alternative hypothesis. The **left panel** shows the posterior and prior; the **middle panel** shows the Bayes factor robustness check, and the **right panel** shows the sequential analysis. With a Bayes factor BF_0__+_ of 41.08, the evidence appears very strong in favor of the null hypothesis as compared to the predictive dissonance hypothesis.

**FIGURE 4:**
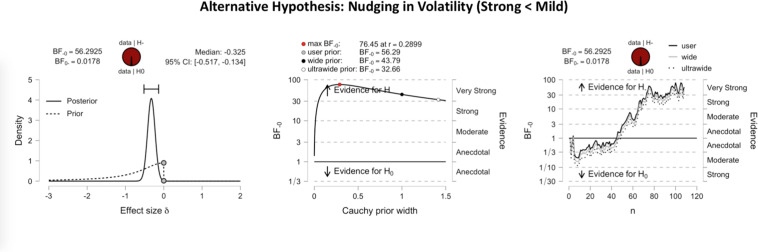
Inferential plots of Bayesian paired-samples *t*-test, with nudging in volatility as the alternative hypothesis. Same format as in Figure 3. With a Bayes factor BF_–__0_ of 56.29, the evidence appears very strong in favor of the nudging-in-volatility hypothesis as compared to the null hypothesis.

Figure 3 shows the inferential plots of a Bayesian paired *t*-test with predictive dissonance as the alternative hypothesis, implying greater validity effect measures for strong than for mild images. With a Bayes factor BF_0__+_ of 41.08, the evidence was very strong in favor of the null hypothesis over the predictive dissonance hypothesis. Conversely, Figure 4 shows the inferential plots of a Bayesian paired *t*-test with nudging in volatility as the alternative hypothesis, implying smaller validity effect measures for strong than for mild images. The Bayes factor BF_–__0_ of 56.29 indicated that the evidence was very strong in favor of the nudging-in-volatility hypothesis rather than the null hypothesis.

## Discussion

Bivalent predictive cues produce an active confirmation bias in a value-based judgment task with naturalistic food images (Ounjai et al., 2018). Here, we investigated the operation of the confirmation bias as a function of the size of the expectation violation. Specifically, we reanalyzed our data set through an item analysis that separated “mild” from “strong” food images, based on their overall ratings across all conditions. Mild images, in case of a mismatch with the valence of the predictive cue, represent a smaller expectation violation than strong images. This moderate versus extreme polarization in the values of the images turned out to modulate the active confirmation bias significantly in the present data. For mild images, we obtained solid cue validity effects, indicating the operation of a confirmation bias, with more positive ratings for appetitive images following a positive predictive cue than following a negative predictive cue, and vice versa. For strong images, we did not obtain significant cue validity effects.

Our findings support the nudging-in-volatility hypothesis, suggesting that the predictive cues are influential predominately for food images that do not have a strongly polarized value. In line with previous findings on the critical aspect of volatility (Massi et al., 2018; Findling et al., 2019), we propose that the mild images have relatively unstable value associations that make them susceptible to the prodding or nudging by predictive cues. Thus, the volatility enables nudging.

Within the framework of active inference, our findings are consistent with the notion that top-down bias is most evident under increased sensory uncertainty (Hohwy, 2017). More specifically, when a stimulus with a volatile affective value follows an unambiguous predictive cue, this would elicit a precision prediction error. The predictive cue itself was categorical: either positive or negative. Yet, a food image with volatile value would be harder to classify. The resulting prediction error then would be imprecise. In terms of active inference and updating, Friston et al. (2016) have discussed in detail how such a precision prediction error, signaled by dopamine, effectively drives down the gain on the prediction error. In other words, less updating will follow from the prediction error because imprecise prediction errors cannot be trusted. The inference is then weighted more by the prior, induced by the predictive cue. The prediction becomes more influential. Hence, in the present paradigm, the confirmation bias occurs particularly under this imprecision, with target images that have volatile affective values.

Subjects might–consciously or unconsciously–turn to the external information, provided by the predictive cue, particularly when they are in doubt about the value of a given food image. Interestingly, this notion of extra information integration under doubt is also compatible with other findings from our laboratory on the cognitive mechanisms underlying the evaluation of food images. Particularly, across two connected studies, Wolf et al. (2018, 2019) found that subjects tended to gaze longer at images for which they felt uncertain about the evaluation, completely against the prevailing notion in the literature that “viewing leads to liking.” Again, the finding of longer gazing at items that are not clearly liked or disliked can be interpreted in terms of volatility. That is to say, volatility appears to be a condition that is more likely to lead to value updating or information integration toward improving the prediction precision.

The present data offer firm evidence against the predictive dissonance hypothesis–at least our current derivation in the present experimental paradigm. To be sure, this in no way discredits the theory of predictive dissonance (Kaaronen, 2018). Rather, we suggest that our present findings introduce a critical dimension to be considered in the theory of predictive dissonance. One way of interpreting the current data would be to flip the perspective and emphasize that strong images, with stable value associations, are impervious to outside influence. Against these strong images, the predictive cues have limited power, likely because the subjects have no particular affective investment in them. The prediction, or “the belief,” implied by the cue has little or no meaning to the subjects in the larger scheme of things. This is very different from other kinds of beliefs and predictions that may be of deep personal importance to individuals (e.g., “Climate change is a hoax”). There may indeed be strong resistance against accepting evidence that challenges a person’s core views, particularly when the evidence is striking and would require a fundamental revision. Thus, we propose that, in the theory of predictive resonance, a critical weight factor in the prediction should be included. Without much weight to the prediction, nudging may work for volatile values, while the predictive information provided by the cues is largely abandoned for images with strongly polarized, stable values.

## Data Availability Statement

All datasets presented in this study are included in the article/Supplementary Material.

## Ethics Statement

The studies involving human participants were reviewed and approved by the Human Ethics Committee of the Faculty of Arts and Science, Kyushu University (Issue number 201711). The patients/participants provided their written informed consent to participate in this study.

## Author Contributions

KO, JH, and JL contributed to the design of the study. KO conducted the data collection for the study, analyzed the data with LS, and prepared the figures. JL wrote the manuscript. All authors reviewed and approved the manuscript.

## Conflict of Interest

The authors declare that the research was conducted in the absence of any commercial or financial relationships that could be construed as a potential conflict of interest.

## Supplementary Material

DATA SHEET S1 Complete data set for the present study, organized in three Data Sheets: Experiment 1, Experiment 2, and Pooled for Bayesian.
